# Biotech MERCOSUR project: an integrated genotyping and phenotyping platform of *Eucalyptus* germplasm for mapping purposes

**DOI:** 10.1186/1753-6561-5-S7-P33

**Published:** 2011-09-13

**Authors:** Susana N Marcucci Poltri, Nuno Borralho, José Carlos Rodrigues, José Elizaul, Mirtha Vera de Ortiz, César Cardozo, Guillermo Salvatierra, Dario Grattapaglia

**Affiliations:** 1 Instituto Nacional de Tecnología Agropecuaria. Instituto de Biotecnología (INTA). De Los Reseros y Dr. Nicolás Repetto s/n°, CP 1686, Hurlingham, Buenos Aires, Argentina; 2 Forestacion S.A. Oficina en Florida. Batlle 843. Esq. Suarez, Montevideo, Uruguay; 3 Instituto de Investigação Científica Tropical (IICT), Centro de Florestas e Produtos Florestais, Tapada Ajuda, 1349-017 Lisboa, Portugal; 4 Desarrollos Madereros S.A. Supercarretera Itaipú Km. 32; Norte – Hernandarias, Paraguay; 5 Facultad de Ciencias Agrarias. Universidad Nacional de Asunción, Paraguay; 6 Centro Multidisciplinario de Investigaciones Tecnológicas / Dirección General de Investigación Científica y Tecnológica, Universidad Nacional de Asunción, Parguay; 7 Garruchos S.A.Estancia Puerto Valle. Ruta Nacional N° 12 - KM 1.282, Corrientes, Argentina; 8 Empresa Brazileira de Pesquisa Agropecuaria, Unidad Recursos Genéticos e Biotecnología. Parque Estação Biológica - PqEB - Av. W5 Norte (final). Caixa Postal 02372 - Brasília, DF, Brazil

## Background

The Biotech MERCOSUR project (85% founded by the European Union) launched in January 2009 and finished in December 2010. The general objective was to establish a regional scientific-technological network to develop a genotyping and phenotyping platform useful for the improvement of *Eucalyptus* in the region. Two advanced genomic strategies (genetic and association mapping) were applied to investigate the genetic basis of the industrial wood formation and energy production of *Eucalyptus* plantations. Up to date, no projects were carried out involving the four MERCOSUR countries altogether. The project brought together scientists from 3 MERCOSUR public research institutes and 2 industrial partners from 4 countries.

The project was possible thanks to the availability of five evaluated field trials of *Eucalyptus*, four segregating populations, a high-density Diversity Arrays Technology (DArT) resource of wide genome coverage and high transferability technology for the genera [1], as well as phenotypic assessment methods of physical and chemical properties of wood using Near Infrared Reflectance (NIR) [2] together with the classical measurements of growth and wood quality tree properties.

The aim of this work is to present the main inputs and results achieved in the project.

## Materials and methods

### Plant material

Open pollinated (OP) progeny trial of *Eucalyptus grandis* planted in Argentina: 188 trees from 132 OP families (1 to 3 trees per OP family) from 13 native stand sites in Australia and 3 Argentinean land races.

Clonal trial of *E. grandis* planted in Paraguay: 123 clonal trees most of them with unknown provenances.

OP progeny trials of *E. globulus* planted in Uruguay: 169 trees from 132 OP families (1 to 8 trees per OP family) from 8 Australian geographic races and one Chilean land race.

OP progeny trial of *E. globulus* planted in Argentina: 134 trees from 129 OP families (1 or 2 trees per OP family) from 8 Australian geographic races and 2 landraces from Chile and Portugal.

Full-sib progeny trial of *E. grandis* planted in Argentina: 130 full-sib trees (see García et al. in this journal).

*E. grandis*× *E. urophylla*: described in [3].

### Genotyping

Over 7,600 clones of DArT *Eucalyptus* microarray were screened.

From 12 to 19 polymorphic simple sequence repeat (SSR) markers [4]were used to estimate diversity parameters as well as inbreeding coefficients for the *E. grandis* and *E. globulus* populations. For map development, more than 300 SSR markers were tested.

### Growth, shape and wood quality tree measurements

Standard methods were used to measure diameter at breast height, total height, stem straightness, pilodyn penetration and basic density for all population analyzed.

### Chemical wood properties for NIR spectroscopy predictions

Lignin contents was measured by Klason method, extractives by an adjusted laboratory protocol, Syringyl:Guaiacyl ratio by analytical pyrolysis.

Pulp yields were calculated based on the oven-dry weight of wood chips charged to the reactor at Kappa number 18.

The NIR spectra were obtained by diffuse reflectance on a Bruker model MPA

### Data analysis

Several tools for data analysis were applied according to the objectives of the different approaches (preliminary association mapping, see Cappa et al.; genetic mapping, see García et al., this journal and [3].

## Results

1. Germplasm characterization with DArT markers in two *E. grandis* and two *E. globulus* populations: between 2,300 and 2,816 DArT (call rate higher than 0.80, and polymorphic for more than 95% of individuals) were included in a preliminary association analysis (Cappa et al. in this volume).

2. Linkage Mapping for QTL detection with DArT (between 1500 and 2000 segregating markers per population), SSR, EST-SSR and candidate genes in *E. grandis* population (García et al., this volume) and in *E. grandis* x *E. urophylla*[3].

3. NIR-PLSR models were developed to assess extractives (ethanol and water) and lignin content (Klason and total) as well as lignin composition (S/G ratio) and pulping yield for *Eucalyptusglobulus* and *E*. *grandis*. All models obtained were at least good enough for screening with RPD above 2 [2]. The RPD obtained varied with trait and species (*E*. *globulus*: extractives-2.3; Klason lignin-3.9; total lignin-3.8; S/G-3.8; pulping yield (K18)-3.5. *E*. *grandis*: extractives-5.9; Klason lignin-6.5; total lignin-3.2; S/G-4.2).

4. Phenotyping analyses: between 2,000 and 16,000 trees were characterized for 2 to 5 traits (diameter at breast height, total height, stem straightness, pilodyn penetration and basic density). This data for all population is available as a product of the project. The phenotypic distribution of all continuous traits was studied. Then, to deal with environmental variation, the data were analysed using a mixed linear model with separable first-order autoregressive residuals for rows and columns (i.e., with a standard spatial analysis).

5. Generation of clonal populations in Argentina and Uruguay for future association mapping studies: two *E. globulus* and one *E. grandis* (*n* > 200 each) for a more precise and thorough evaluation of phenotypes are being planted.

6. Origin assign for clone trees of unknown provenances. This was possible due to the combination of DArT markers analysis with population structure approaches. More than 50 clones were assigned to defined clusters.

7. Training of the participants involved: 4 lab training and 2 courses were developed for different participants of the project.

## Conclusions

The genotyping and phenotyping platform was successfully established and encourage the collaboration between the participants of the different countries. Likewise different strategies have established the basis for developing new works in the near future using the resources and tools generated in the context of the Biotech MERCOSUR project.

## References

[B1] Sansaloni CP Petroli CD Carling J Hudson CJ Steane DA Myburg AA Grattapaglia D Vaillancourt RE Kilian A A high high-density Diversity Arrays Technology (DART) microarray for genome genome-wide genotyping in Eucalyptus Plant Methods 2010 6 16 10.1186/1746-4811-6-16 20587069 PMC2903579

[B2] Williams PC Sobering DC Comparison of commercial near infrared transmittance and reflectance instruments for analysis of whole grains and seeds J. Near Infrared Spectrosc 1993 25 32

[B3] Petroli CD Sansaloni CP Kilian A Steane DA Myburg AA Pappas GJ Faria DA Vaillancourt RE Grattapaglia D A high-density sub-centiMorgan integrated DArT/microsatellite genetic linkage map for species of *Eucalyptus* based on 2,980 markers Resumos do 56° Congresso Brazileiro de Genética 2010 http://web2.sbg.org.br/congress/sbg2008/pdfs2010/GP159-34030.pdf

[B4] Brondani RP Williams ER Brondani C Grattapaglia D A microsatellite-consensus linkage map for species of *Eucalyptus* and a novel set of 230 microsatellite markers for the genus BMC plant biology 2006 6 20 10.1186/1471-2229-6-20 16995939 PMC1599733

